# Autologous Neurosensory Retinal Transplantation: A Report of Three Cases

**DOI:** 10.18502/jovr.v16i1.8252

**Published:** 2021-01-20

**Authors:** Ogugua Ndubuisi Okonkwo, Adekunle Olobola Hassan, Toyin Akanbi

**Affiliations:** ^1^Eye Foundation Hospital, Apo. Abuja, Nigeria; ^2^Eye Foundation Retina Institute, Ikeja. Lagos, Nigeria

**Keywords:** Autologous Transplant, Macular Hole, Neurosensory Retina, Vitrectomy

## Abstract

**Purpose:**

To present the anatomical and functional outcomes of autologous surgical transplantation of a free neurosensory retinal graft in three cases of recurrent and chronic full thickness macular hole (MH).

**Methods:**

A retrospective case series, reporting the profile, preoperative presentation, surgical technique, and postoperative outcome of three consecutive eyes of three patients who had autologous retina transplantation (ART) surgery for recurrent and chronic MHs, and had a minimum of six months follow-up. The technique involved excision of a free neurosensory graft after laser demarcation of the harvest site. The graft was slid under perfluorocarbon liquid (PFCL) into the MH. A five-day tamponade with PFCL was used to secure the graft within the MH and then exchanged with air.

**Results:**

The patients were one female and two males aged 60, 44, and 67 years, respectively. All eyes had successful surgery. Postoperative vision improved from 6/36 to 6/18 in patient 1 and remained same as preoperative vision in the other two eyes. No eye lost vision postoperatively. The main complication of surgery was occurrence of retinal and vitreous hemorrhage in one eye (this did not appear to jeopardize the outcome) and retraction of graft tissue in two eyes.

**Conclusion:**

ART appears to be a safe and effective treatment for difficult MHs. Our results are comparable to previous studies. Short-term use of PFCL can be useful to secure the graft within the MH. Methods of improving visual function should be the focus of further research in this promising area.

##  INTRODUCTION

There are few reports on the techniques for autologous retinal transplantation (ART). ART has shown to be promising for the treatment of recurrent, chronic, and myopic macular hole (MH).^[1,2]^ It involves the harvest of a free patch of neurosensory retina from an extra macular site and placing this graft within the MH. It is hoped that the piece of neurosensory retinal tissue will remain within the hole and eventually get integrated into the surrounding retina and that the surviving graft tissue will provide vision. Initial cases reported dislocation of the neurosensory retinal patch from within the MH intra- and postoperatively.^[3]^

We report our experience of three consecutive eyes that had ART with short-term perfluorocarbon liquid (PFCL) tamponade and a minimum of six months follow-up.

##  METHODS

### Surgical Steps For ART

Our technique was a standard 23G unimanual vitrectomy, for all three cases. The Constellation vitrectomy unit (ConstellationⓇ, Alcon, Fort Worth, TX, USA) was used. PFCL injection was followed by a laser demarcation of the chosen site for the free graft harvest. Neurosensory retinal tissue was excised within the laser-demarcated area, using a 23G intraocular vertical scissors. The free graft was then slid under the PFCL into the MH with the use of intraocular forceps. The harvested piece of retinal tissue was teased open and spread over the MH (but not tucked into it) to cover the MH using the soft edge of a silicone-tipped cannula. PFCL was left in the eye for five days to secure graft stability and prevent graft dislocation from the MH. On the fifth day, air-fluid exchange was performed.

There was no positioning of the patient within this five-day period. The patient was allowed to assume any face or head position they chose during this five-day period.

The fluid-air exchange was performed in the operating theatre and took approximately 15 min to do. The procedure involved the use of a 23G system. Air was infused into the eye, while silicon-tipped cannula was utilized to aspirate the PFCL. After all the PFCL bubble was removed, a fluid rinse of the vitreous cavity using balanced salt solution (BSS) was done to ensure that all possibly trapped bubbles of the PFCL in the vitreous base and elsewhere were rinsed out into the vitreous-filled BSS, and this was removed with a repeat fluid-air exchange. This fluid rinse was repeated several times to ensure the vitreous cavity was free of PFCL droplets. An irrigation of the anterior chamber (AC) with saline was also performed to ensure that there were no PFCL bubbles in the AC.

All the eyes were commenced on frequent topical steroids (Pred Forte eye drops) and antibiotics as a standard protocol. All three patients gave a written informed consent before the procedure.

##  RESULTS

There was one female and two males aged 60, 44, and 67 years, respectively. Patient 3 was diabetic, but achieved a good control of blood sugar. The other two patients had no systemic comorbidities. All three eyes had successful surgery with retention of the free neurosensory retinal graft within the MH. Integration of the graft with surrounding retinal tissue was evident on OCT after three months in patient 1, as shown in Figure 3. In patients 2 and 3, the free neurosensory graft plugged the MH as seen in Figures 5 and 8, which show the appearance of tissue plugging the MH. Postoperative visual acuity improved from 6/36 to 6/18 in one eye and remained same as preoperative vision in two eyes. No eye suffered a loss of vision. All three eyes were pseudophakic and maintained normal intraocular pressures postoperatively. There was no complication or excessive inflammation noticed from the five-day use of PFCL as tamponade. Full details of the cases are described below.

**Figure 1:**
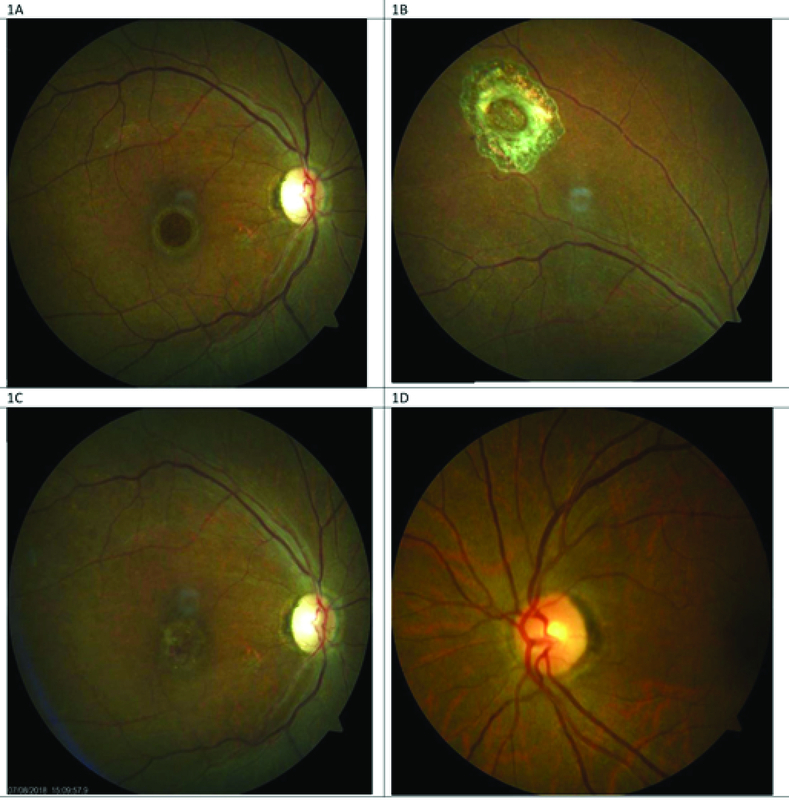
(A) Right eye preoperative fundus photograph showing the appearance of a recurrent macular hole occurring in patient 1; Pre-ART surgery. (B) Laser scar surrounding the site of neurosensory retinal harvest, anterior to the superotemporal arcade. (C) Post-ART surgery fundus photograph showing appearance of the free neurosensory retinal graft over the macular hole. (D) Normal-appearing left eye of the same patient.

**Figure 2:**
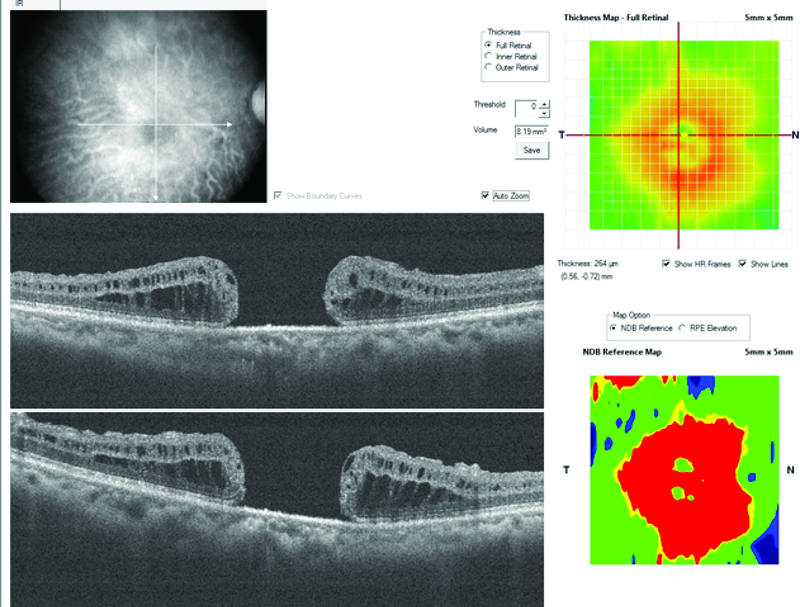
Patient 1; preoperative OCT cross-line horizontal and vertical scans of the macular hole.

**Figure 3:**
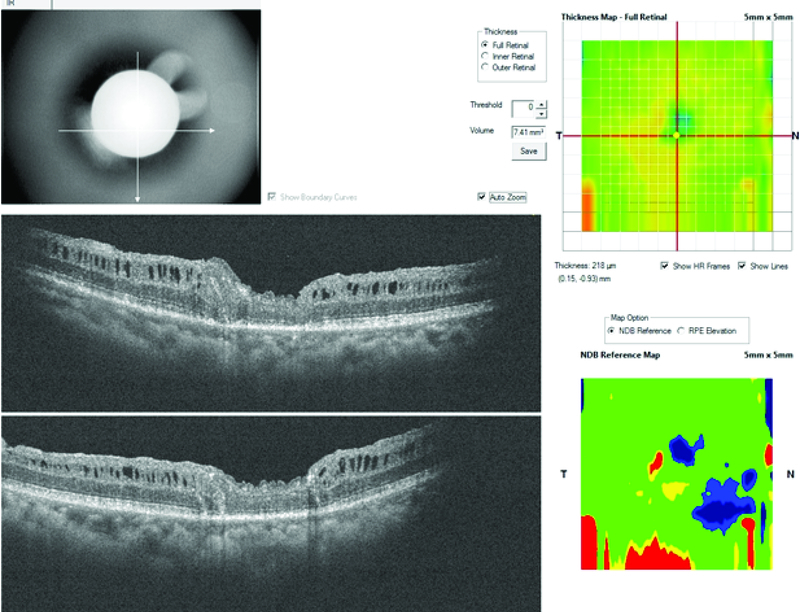
Patient 1; postoperative OCT cross-line horizontal and vertical scans showing integration of the ellipsoid zone of the retina graft tissue with the adjoining host retinal tissue.

**Figure 4:**
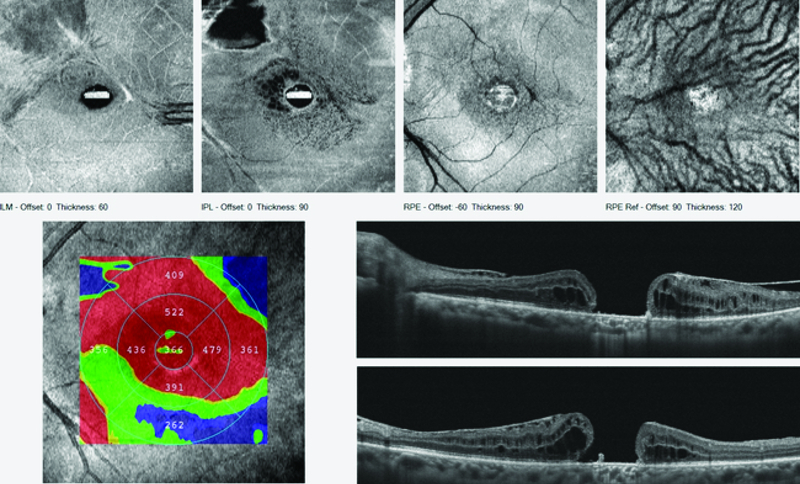
Patient 2; preoperative OCT cross-line horizontal and vertical scans of the macular hole; plus enface images of the macular hole.

**Figure 5:**
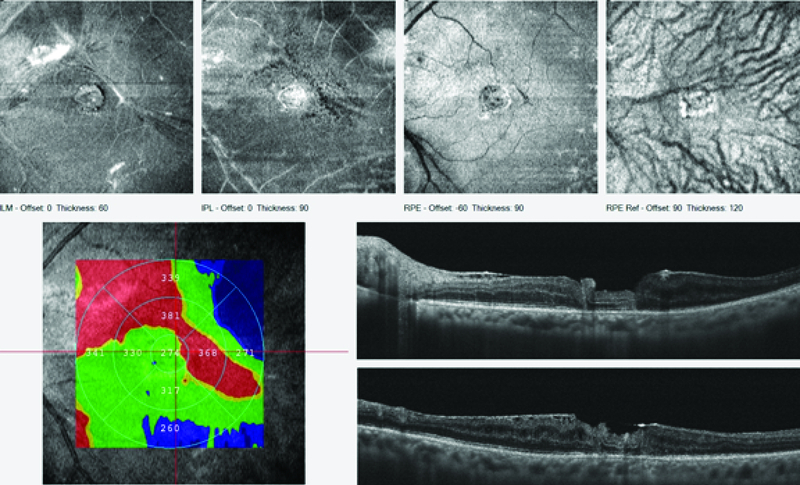
Patient 2; early postoperative OCT cross-line horizontal and vertical scans with graft tissue plugging the macular hole. The intraretinal architecture is preserved.

**Figure 6:**
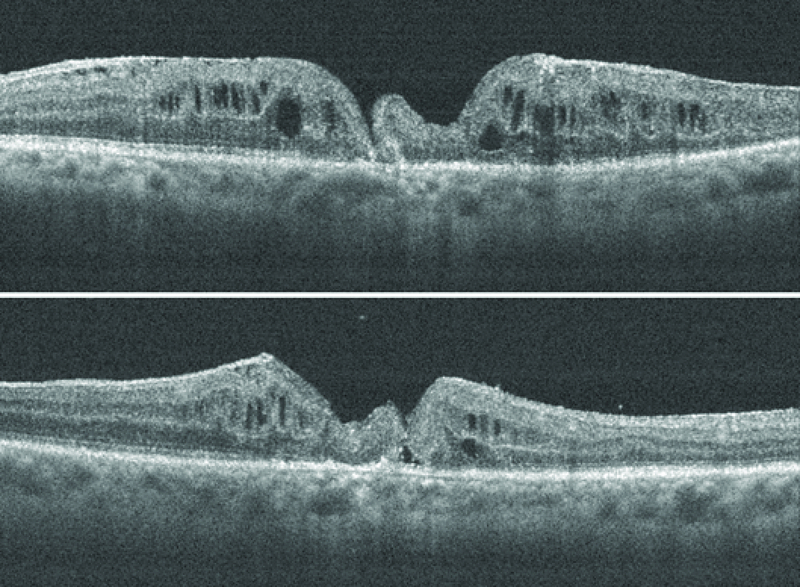
Patient 2; later postoperative OCT cross-line horizontal and vertical scans with retraction of the retinal graft. There is a gap between the edge of the graft and the macular hole.

**Figure 7:**
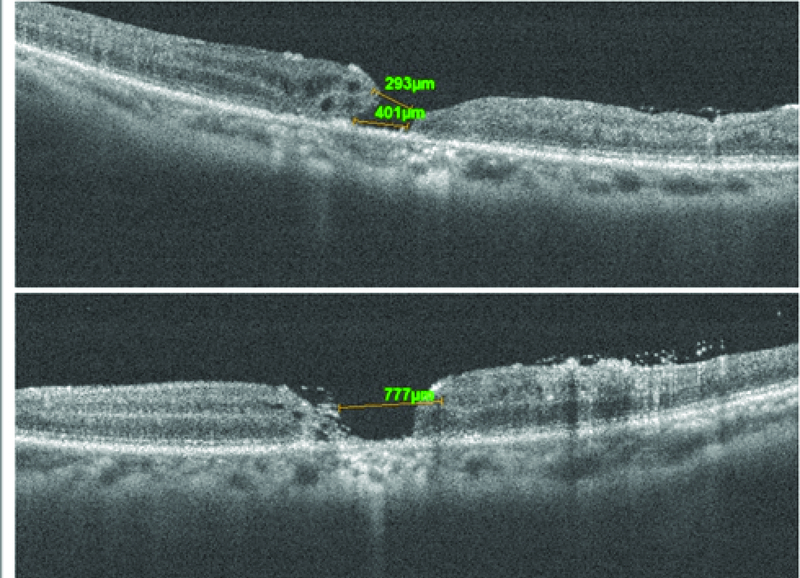
Patient 3; preoperative OCT cross-line horizontal and vertical scans of the macular hole. Emulsified silicon oil is present on the retinal surface and within the macular hole.

**Figure 8:**
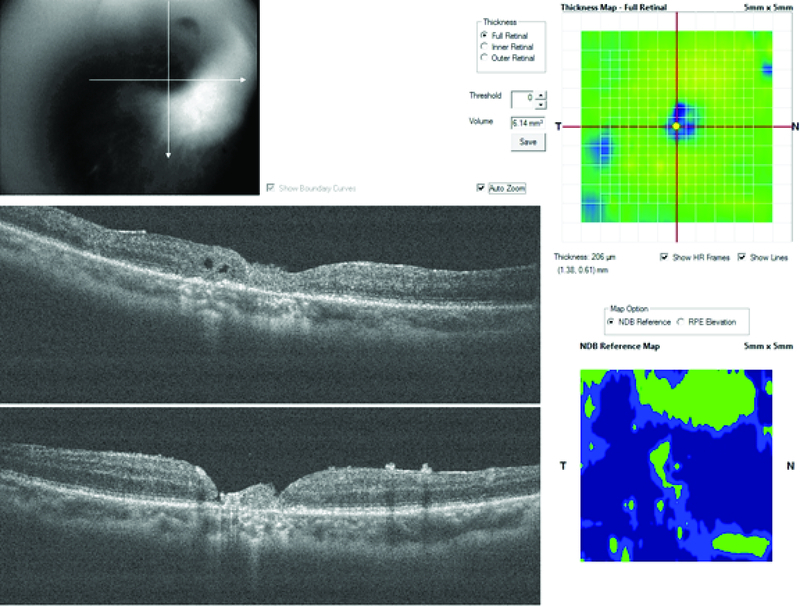
Patient 3; postoperative OCT cross-line horizontal and vertical scans showing retraction of the retinal graft and no preservation of the intraretinal architecture.

### Complications

The only intraoperative complication of note, which occurred in patient 3, was intraoperative retinal hemorrhage that happened during the time of neurosensory retina harvest and continued as a postoperative vitreous hemorrhage. He had been on anticoagulants prior to the surgery. This hemorrhage was limited by the PFCL and an adjacent fresh site of harvest was then chosen, and the procedure was completed as planned. Two eyes had retraction of graft tissue, which was evident on postoperative OCT.

### Case 1 

The first case is of a 60-year-old female, who had a reopened right eye MH after an initial internal limiting membrane (ILM) peel procedure in November 2014. Visual acuity was 6/36, and she complained of a persistent central scotoma due to the recurrent MH (Figure 1A). The pre-ART MH base diameter was 1200 microns (Figure 2). ART surgery was performed in July 2018. Site of neurosensory retinal harvest was anterior to the superotemporal arcade (Figure 1B). Post ART surgery, visual acuity improved to 6/18 at two months postoperative visit and had remained so till her last clinic visit in December 2019. The MH was closed, and the retinal graft remained within the MH (Figure 1C). Her left eye macula was normal (Figure 1D). There was preservation of some of the ellipsoid zone (EZ) in the free retinal patch, and this appeared to be continuous with the EZ of the adjacent host retina as seen on OCT (Figure 3).

### Case 2 

The second case is of a 44-year-old male, who presented with a chronic total retinal detachment and proliferative vitreoretinopathy of greater than six months duration, with multiple peripheral retinal breaks and a MH. Right eye was blind from rubeotic glaucoma.

He had a vitrectomy with silicone-oil tamponade in January 2017 followed by removal of the silicone oil in August 2017 with successful retinal reattachment. He regained a postoperative vision of 6/36, however, the MH persisted as shown in the OCT image (Figure 4).

The pre-ART MH base diameter was 1060 microns. ART surgery was performed in January 2019. Site of neurosensory retinal harvest was anterior to the inferotemporal arcade.

Post ART surgery, visual acuity remained 6/36, however, he gave a subjective impression of visual benefit since he claimed to now see facial details better than preoperatively (including facial marks which he could not see prior to ART surgery).

There was anatomical MH closure, with retention of the retinal graft within the MH in the early postoperative period as seen on OCT (Figure 5). However, there were persistent intraretinal cystic spaces. At the third postoperative month, the graft tissue was noticed to have retracted as there was now a gap between the edge of the tissue graft and the edge of MH as seen on OCT (Figure 6). Despite this, the outer retina was noted to be present in the graft tissue on OCT (Figures 5 and 6).

### Case 3

The third case is of a 67-year-old male who presented with a chronic MH and emulsified silicone oil in the right eye (Figure 7). He had a history of having had combined vitreoretinal and cataract surgery in November 2014 during which silicone oil tamponade was used. Visual acuity was 6/36. The MH base diameter was 790 microns and there was emulsified silicone oil within the MH as seen on OCT.

Silicone oil removal combined with ART was performed in January 2019. The site of neurosensory retinal harvest was anterior to the inferotemporal arcade. Intraoperatively, at the time of graft tissue harvest there was a significant retinal hemorrhage. This hemorrhage occurred because the patient was on anticoagulant therapy prior to surgery. The hemorrhage was however limited by the PFCL. This hemorrhage necessitated the abandoning of the harvest site and moving to an adjacent site. Postoperative vision remained 6/36. Postoperative OCT revealed that the retinal graft remained within the MH as shown in Figure 8. However, retraction of the graft as happened in patient 2 was evident, and outer retinal layers or intraretinal structures were not preserved.

##  DISCUSSION

Grewal and Mahmoud first reported the successful transplantation of extramacular retinal tissue into a refractory MH, with good anatomical and functional outcome.^[1]^ Prior to this, several authors have published works on transplantation of retinal pigment epithelium for the treatment of neovascular age-related macular degeneration (AMD).^[4,5,6]^ Since this first report, there have been few case reports on the use of ART for treatment of difficult to treat MHs, such as those associated with retinal detachment.^[7,8]^ Recently, an international collaboration published the largest series on the use of ART for the repair of refractory, large MHs.^[9]^ The findings by this group could provide a yardstick against which future outcomes can be measured. Our consecutive series adds to the growing number of cases and seems to agree with current reports. We found that ART can be performed with relative safety and that it was effective for achieving anatomical MH closure. However, improvements in visual acuity are possibly not yet optimized and may revolve around graft size and harvest site.

Graft size may be an important factor as it was for patients 2 and 3; there was a postoperative retraction of the graft tissue with a reduction in graft size. This suggests that the size of the free retinal graft should be larger than the MH to ensure MH closure even after the anticipated graft retraction, as was suggested in the original report.^[1]^

Improving visual acuity remains a challenge. In our study, one eye had a Snellen acuity improvement in vision, but vision remained the same in the other two eyes. In the collaborative study, vision remained unchanged in 41.5% of eyes and worsened in 21.9% of eyes. None of our patients had a worse postoperative vision, including patient 3 who suffered intraoperative hemorrhage. However, our sample size is much smaller than the reference study. Understanding the functionality of the graft is important to be able to determine visual outcomes in future. Microperimetry can be a useful tool in assessing functionality of the graft tissue, when testing response to light. Other studies reported retinal graft tissue response to microperimetric light testing, but we were not able to perform this in our study.^[2,9]^

In terms of complications, the major postoperative complication encountered was in patient 3 who suffered a retinal and vitreous hemorrhage. The collaborative study also reported one case of the vitreous hemorrhage. Our patient had been on anticoagulants. We therefore recommend considering discontinuation of anticoagulant use before performing this procedure. In our patient, this hemorrhage was limited by the PFCL. Stopping and limiting intra- and postoperative hemorrhage is another useful function of PFCL, as was demonstrated, in this case.^[10]^ In all three cases, PFCL was used as tamponade for only five days and was then replaced with air. No complications of PFCL tamponade were noticed. In particular, no exaggerated intraocular inflammation due to the use of PFCL was seen within the period of follow-up. Short-term use of PFCL tamponade has been reported previously without significant complications and was also used in the collaborative study.^[9,11,2]^ PFCL served as a good tool to ensure the free graft covered the MH. Furthermore, it is possible that the PFCL may provide oxygen for graft survival in the early stages of transplantation.^[13]^

To conclude, this study appears to concur with previous reports, suggesting that ART is a relatively safe technique in the management of refractory, chronic MHs. The visual outcome, which may be unpredictable, requires further research to determine optimum graft to host (MH) size and functionality at the macula.

##  Financial Support and Sponsorship 

Nil.

##  Conflicts of Interest 

There are no conflicts of interest.
